# Case report: Lower eyelid Reconstruction with the rotation flap pedicled by orbicularis oculi muscle

**DOI:** 10.3389/fsurg.2022.915124

**Published:** 2022-08-08

**Authors:** Mengying Duan, Chao Yue, Jianzhong Peng

**Affiliations:** Department of Dermatologic Surgery, Hangzhou Third People's Hospital, Hangzhou, China

**Keywords:** rotation flap pedicled, orbicularis oculi muscle, lower eyelid defects, reconstruction, surgical procedure

## Abstract

**Background:**

Loss of eyelid tissue can be caused by trauma, congenital defects or tumors. Eyelid reconstruction is complicated and challengingly difficult because of the complex anatomy of the eyelid. Several types of surgical procedures for the reconstruction of eyelid defects are available.

**Objective:**

To describe reconstruction of lower-eyelid defects using a rotation flap pedicled by the orbicularis oculi.

**Methods:**

Fourteen patients (mean age = 67 years old; ages range of 53–86 years old) who suffered from tumor excision from the lower eyelid were treated by the method.

**Results:**

The mean duration of follow-up was 14 (range, 12–16) months. Ectropion, abnormal eyelid position and donor-site morbidity were not observed during follow-up.

**Conclusion:**

A rotation flap pedicled by the orbicularis oculi can be a good choice for single-stage reconstruction of lower-eyelid defects.

## Introduction

Loss of eyelid tissue can be caused by trauma, congenital defects, or tumors (1). Tumor excision accounts for a considerable proportion of eyelid reconstructions. Twenty-five percent of eyelid tumors are malignant (2), and up to 14% eyelid tumors attributes to basal cell carcinomas emanated from the periorbital area, of which 90% were involved the eyelids (3). Squamous cell carcinoma is uncommon, but is often found on the lower eyelid and aggressive (4). Due to the complex anatomy of the eyelids, changes in one structure can have an impact on other anatomic units. As a result, the paramount goal in eyelid reconstruction is aesthetically optimal restoration of function and bilateral symmetry in a minimum of surgical morbidity.

A good knowledge of anatomy and function in the periorbital area is essential to the successful reconstruction of the eyelids. The latter plays an important role in protecting and lubricating the eyes as well as drainage of the lacrimal system (1, 5).

The eyelid is comprised of the anterior lamella and posterior lamellae. The latter is lined with the tarsus and conjunctiva. The anterior lamella consists of muscle and skin (3). The orbicularis oculi (OO) is supplied with a vascular arch which is created by the branches of the ophthalmic artery perforating the extremities of the tarsus (6). Numerous surgical methods have been used to reconstruct a defect in the eyelid, aiming at the minimal deformation of this structure.

As for defects of the lower eyelid, flap reconstruction can offer more effective treatment than graft placement. Therefore multiple flaps have been used by surgeons: nasolabial, temporal artery “island”, myocutaneous and so on (6–8).

The paper studies the rotation flap pedicled by the OO. This strategy generates reconstruction of a lower-eyelid defect with tissue composition which similar to that of a healthy eyelid. But the reconstruction is also faced up with many challenges. Application of a rotation myocutaneous flap in this way has not been reported previously.

## Materials and methods

### Ethical approval of the study protocol

The study protocol was approved by the Institutional Ethics Committee of the Hangzhou Third People's Hospital.

**Figure 1:**
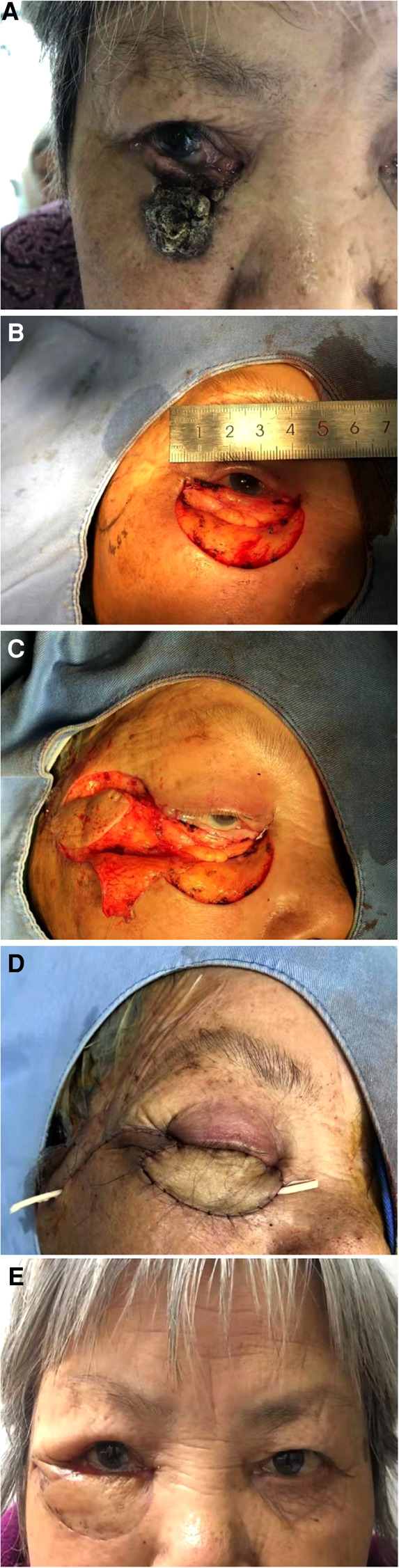
(**A**) Preoperative view of the patient. The patient was diagnosed with basal cell carcinoma by pathological examination. (**B**) The tumor was excised with safe edge by Mohs. The size of the defect was approximately 4.0*3.5 cm² and the tarsus and mucous membrane was reserved. The flap was designed. (**C**) The flap was elevated and prepared to close the defect. (**D**). The flap was sutured to the defect on the lower eyelid. (**E**) Reconstruction follow-up after 12 months. The patient was satisfied.

The medical records of 14 patients (eight men and six women; mean age = 67 years old; age range of 53–86 years old) who have been treated lower-eyelid reconstruction with a rotation flap pedicled by the OO from September in 2020 to January in 2021 were kept (Table 1). The same surgical strategy was applied to all patients. What's more, there were no technical differences in the surgical procedure. The pre-, intra-, and postoperative images of patients were reviewed (Figure 1).

**Table 1:** Patient data.

Patient No.	Age/Sex	Etiology	The size of skin defect(cm)
1	76/F	BCC	3.5*2.2
2	53/M	BCC	2.7*2.0
3	69/F	BCC	3.0*2.1
4	70/F	BCC	3.7*2.5
5	75/M	BCC	3.5*2.3
6	56/F	BCC	3.5*2.5
7	56/M	BCC	2.8*2.3
8	86/M	BCC	3.9*3.0
9	80/F	BCC	3.8*3.0
10	75/M	BCC	3.0*2.2
11	78/M	BCC	3.8*3.0
12	81/M	BCC	4.0*3.5
13	73/F	BCC	3.8*2.8
14	66/M	BCC	3.5*2.8

F, female; M, male; BCC, basal cell carcinoma*.*

Lesional tissue wasn't excised until clear safe margins were achieved. Frozen sections were used to ensure that lesional tissues were completely cleared by Mohs micrographic surgery. The strategy for defect reconstruction was based on transfering of a rotation flap pedicled by the OO to the defect site. The flap was marked on skin at the lateral border of the defect (which was between the outer canthus and the sideburn). The vertical distance from the lower eyelid was defined as the width of the defect . The distance of the defect parallel to the palpebral margin is defined as the length of the defect. The length of the defect corresponded to the flap. The width of the defect was 1.5-times of the width of the flap. Maintaining some tension prevented flap from bloating. With regard to the blood supply, the skin incision was extended to the deep fascia, and an adequate volume of subcutaneous fat was included in the flap. On account of rotation, the pedicle (which was close to the outer canthus) was designed to be lateral to the flap. However, to repair the lower eyelid, the tip of the flap was dissected as thin as possible above the deep fascia layer. The pedicle had to contain the OO and cover ≥50% of the flap to ensure a maximal blood supply. The pivot point for flap transposition was at the medial end of the skin incision. The flap was transferred into position by rotating it 180°around the pivot point, after which the donor site was closed without producing a conspicuous scar. Oral mucosa, inner prepuce plate or inner mucosa of labia minora can be lined if the tarsal plate and the mucous membrane are involved, which can reduce the friction irritation of the inner flap to the eyeball. A suture could be used to fix the flap on the orbital periosteum to avoid ectropion. A temporary tarsorrhaphy with 7/0 absorbable suture material was used to stabilize the flap. The donor site was closed by bilateral advancement of the surrounding tissue. After appropriate pruning, a rotation flap pedicled by the OO was just to cover the lesion. Stitches were removed after 4 days. Patients were asked to attend outpatient clinics at monthly intervals after the procedure.

The mean duration of postoperative follow-up was 14 (range, 12–16) months. All flaps survived well and the skin regained its texture and color gradually. All patients were satisfied with the treatment. Deformation of the lower eyelid was not observed and scarring was not obvious. Major complications (abnormalities in eyelid position, corneal irritation, flap contraction) were not seen in any patient. Neither flap necrosis nor ectropion were observed. All patients could open or close their eyes freely. Even the length of the defect is the same as the whole length of the lower eyelid, we can also use the flap to reconstruct the lesion. In other words, the flap can fix defect as long as the lesion size is no more than the size of the lower eyelid. In short, excellent functional and esthetic results were obtained.

## Discussion

Lower-eyelid tissues are thin and elastic, and reconstruction of this area is challenging. In addition to complete removal of the lesion, attention should be paid to achieving cosmetic and functional outcomes of the eyelid. If the postoperative scar needs to be minimal and inconspicuous, a rotation flap may be the optimal choice for reconstruction.

In the study conducted by Yousefiazar and his colleagues (7), modification of a Tessier nasojugal flap was applied to reconstructing a medium-sized (30%–60%) medial-side eyelid defect. A nasolabial flap was designed to transfer to the defect site, in which a tunnel under the OO was produced and the dermal tail was stretched through the tunnel by suturing it to the lateral canthus to prevent ectropion. The advantage of this type of surgery is avoiding ectropion and achieving a satisfactory cosmetic outcome. However, tunnel creation is inconvenient compared to the use of a rotation flap pedicled by the OO.

Yang and Zhao harvested island flaps with a vascular pedicle (8). After blood vessels had been detected by a Doppler ultrasound probe, an island flap pedicled with a superficial temporal artery was transferred to the lesional site *via* a tunnel dissected above the zygomatic arch. Despite its advantages, their method is limited because of the vascularity of the underlying subcutaneous facial tissue. Venous congestion cannot be avoided readily as a result of this deficiency of the venous system. Furthermore, the flap requires an appropriate tunnel width and length of vascular pedicle: the blood supply to the flap will be affected if the requirements are not met.

Tirone et al. described application of a myocutaneous flap araised from the upper eyelid (6). They showed skin strips in the upper eyelid to be well-nourished because the blood supplied by the muscle and the skin of the entire upper eyelid could be pulled with an extremely narrow pedicle with the pivot point outside the eyelid. However, the drawback of the flap was poor venous drainage, especially for the elder.

In the procedure described by Barin and Cinal, a musculocutaneous flap based on the OO was designed to repair lower-eyelid defects (9). A chondrocutaneous graft was prefabricated under a pre-septal portion of the OO, and then a musculocutaneous island flap was elevated with the graft after 3 weeks. The flap was incised and transposed to the defect. But the application need a second stage and the skin laxity of the upper eyelid has to be examined. The upper eyelid should be sufficiently lax to ensure that the cutaneous island is available for the flap. The applicability of this method may be limited by the amount of excess eyelid skin available. The method is beneficial to the older patients yet may not be suitable for the young.

Reconstruction of a lower-eyelid defect can be undertaken with a versatile temporoparietal fascia flap (10). Even though the flap is more suitable to the young patients, the eyelid margin is often scarred with the loss of lashes. Reconstruction of a full-thickness medial-half of a lower-eyelid defect can be repaired by a nasojugal flap or a Tessier flap with a chondromucosal graft.

Ko et al. applied a “flip-back” myocutaneous advancement flap to periocular reconstruction (11). However, a suture must be placed between the closed flap to link the outer canthus with the orbital periosteum, thus preventing ectropion, whose principle is the same as “dog ear” of tissue yet superior to it since the “dog ear” of tissue must be excised.

Another method that applies adjacent tissue is an OO myocutaneous island flap elevated from the upper eyelid for canthal reconstruction. The method, introduced by Han et al., avoids crispation of the deformation and punctum of the lacrimal apparatus (12). However, the method has only limited applicability if the defect is too large because the residual OO is essential to the reconstruction.

Albanese et al. used a modified cheek-advancement flap to reconstruct a defect of the medial lower eyelid (13). The procedure causes the face to tighten, bulk at the medial canthus and collapse at the lateral canthus.

A musculocutaneous flap has five main advantages. In the first place, the reconstructed eyelid has similar full-thickness eyelid tissue along with an integrated eyelid margin, so the functional outcome is good. Secondly, it is a one-stage operative procedure. Thirdly, the recipient part of the eyelid maintains an adequate blood supply, thereby reducing the risk of flap necrosis. Fourthly, an abundance of tissue can be used to work with. Finally, complications (e.g., eyelid depression, ectropion) are not observed. However, punctilious and aborative dissection are required for the pedicle of the flap to be reserved.

## Conclusions

We described patients with lower-eyelid defects treated with a rotation flap pedicled by the OO. The length of the flap corresponds to the defect and the width of the flap is approximately 2/3 times of the width of the defect, which effectively reduces the wound area. What's more, the lower edge of the defect is lifted and fixed with periosteum. At the same time, the flap is designed to hide the crow's feet under the Langer's line principle. The rotation flap required simple care and caused less necrosis and swelling in contrast to defects repaired by other reconstructive methods. Our method maximized skin color and vascular match yet minimized wastage of periocular tissue, shortening the operative time. Complications were minimal and the long-term cosmetic results were excellent. The application of a rotation flap pedicled by the OO is novel, reliable, and consistently effective in remedying challenging lower-eyelid defects.

## Data Availability

The datasets presented in this article are not readily available because of ethical/privacy restrictions. Requests to access the datasets should be directed to the corresponding author.
